# Temperature effect on cable force of a special-shaped tied-arch bridge

**DOI:** 10.1016/j.heliyon.2022.e11253

**Published:** 2022-10-26

**Authors:** Liming Zhu, Tailei Chen, Lingkun Chen, Xiaojian Han, Wonsun King, Lu Wang, Chencheng Zhai, Yuan Tian

**Affiliations:** a College of Transportation Science & Engineering, Nanjing Tech University, Nanjing, 211800 Jiangsu China; b College of Architecture Science and Engineering, Yangzhou University, Yangzhou, 225127, Jiangsu, China; c Department of Civil and Environmental Engineering, University of California, Los Angeles, 90095, CA, USA; d School of Civil Engineering, Southwest Jiaotong University, Chengdu, 610031, Sichuan, China; e College of Civil Engineering, Nanjing Tech University, Nanjing, 211800, Jiangsu, China; f Department of Construction Engineering, Chaoyang University of Technology, Taiwan, 413, China; g Transportation Technology Development Promotion Center, China Academy of Transportation Sciences, Beijing, 100029, China

**Keywords:** Temperature, Special-shaped tied-arch bridge, Cable force, Monitoring test

## Abstract

In this study, on-site monitoring was used to obtain the temperature field distribution and cable force values of the bridge at various times, the temperature change pattern of the bridge elements, the temperature sensitivity of the cable force of different parts of the bridge, and the trend of the cable force under temperature load were all analyzed. The static response of the wires was studied under various operating circumstances. The total cable force of the bridge is inversely related to the temperature rise and fall, and the temperature sensitivity of the cable force in different parts of the bridge is different under the same temperature effect. The change in cable force is uneven. They vary according to the loading circumstances. This project's cable force changes are the most substantial and consistent with observed circumstances and should be appropriately considered in comparable projects. Thermal impacts must be addressed in strand cable safety design.

## 1 Introduction

**Figure 1:**
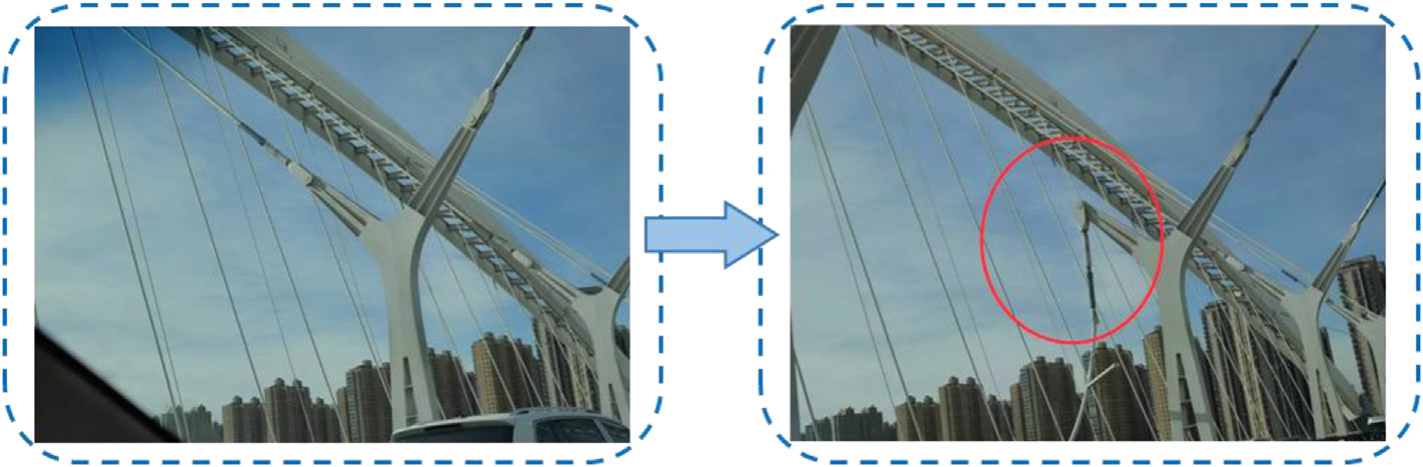
Photos of the accident scene.

The unique-shaped, tied arch bridge has beautiful lines and integrates transportation and ornamental functions. However, its stress is more complex than the conventional tied-arch bridge. As one of the main load-bearing components of a tied-arch bridge, the cable force directly affects the stress state of the bridge. An arch-beam combined special-shaped bridge located in Taiyuan, as shown in Figure 1, after 11 years of opening, an inclined suspender suddenly broke, resulting in the poor overall safety of the bridge, which needs closed traffic inspection and maintenance.

The bridge structure bears loads of vehicles and pedestrians, and its internal force is affected by temperature load. Temperature changes slowly in average weather conditions, limited to a relatively narrow range. When the bridge experiences extreme weather events such as cold waves, heat waves, lightning, and tropical cyclones, unlike average weather, the cyclic variation of ambient temperature will cause large deformation of the bridge, which may exceed the deformation caused by traffic or wind load [1]. Quasi-static responses related to temperature need to be determined and subtracted from the overall measurement to detect undesirable deformations resulting from structural anomalies or deterioration [2, 3, 4]. The temperature effect of bridges has attracted significant attention in the bridge field. To solve this problem, Ji et al. [5] proposed that the cable temperature is inversely proportional to the fundamental frequency. In order to obtain a more accurate cable fundamental frequency, the influence of temperature change and traffic flow should be avoided. Zhao et al. [6] discussed the relationship between temperature and cable force under different initial tensions and proposed that specific particular evident the influence of cooling on cable force will be more evident than that of heating in a certain initial tension range. The temperature difference significantly, the more pronounced cable force and frequency change. Pu et al. [7] analyzed the temperature effect of a cable-stayed bridge from the aspects of the temperature gradient of the main beam, the temperature gradient of the bridge tower, and the structural temperature difference, and proposed that the temperature change of the cable itself had a significant influence on the cable force.

Changes in ambient temperature can cause significant deformation of long-span suspension bridges. The mechanism of midspan deflection and tower top horizontal displacement caused by the temperature of the suspension bridge is studied, and the general analytical solution of thermal response of ground-anchored suspension bridge is given. The temperature-induced mid-span deflection can be determined by the superposition of the deflections caused by different temperature changes in the main span cable, the side span cable, and the tower, while the horizontal displacement at the top of the tower is a combination of the thermal effects of the side span cable and the tower. It is found that cable temperature plays a leading role in mid-span deflection and horizontal displacement of tower top [8]. In order to ensure the cable tension adjustment of concrete cable-stayed bridge smoothly, Ma et al. [9] proposed a new cable tension adjustment technology considering the temperature difference between steel beams. T; the cable tension adjustment process is accurate and efficient because of the vertical displacement of the middle section of the main span as a sensitive structural behavior. Ma et al. [10] studied the influence of ambient temperature on the static and dynamic performance of the cable and proposed a method to identify the cable force considering the environmental temperature fluctuation accurately. Zhang et al. [11] showed a linear relationship between structural deformation and temperature variation based on the analysis of thermal effects on three-tower suspension bridges with unequal spans. The span length variation caused by the bending deformation of the bridge tower significantly influences the elevation temperature sensitivity coefficient of the main cable. This effect can offset the span of thermal shrinkage or expansion on both sides, and the superposition of these two effects occurs in the primary and secondary main spans. Zhang et al. [12] has similar conclusions. The results show that the deformation of a suspension bridge is linear with temperature. The construction state and bending deformation of the tower greatly influence the elevation temperature sensitivity coefficient of the main cable. The bending deformation of the tower increases and decreases the temperature sensitivity coefficient of the main span and side span cable elevation, respectively. The temperature sensitivity coefficient of the mid-span elevation of the main cable is negatively correlated with the span length. The maximum temperature sensitivity coefficient of the central cable clamp installation position appears near the quarter of the main span. Zhu & Meng [13] proposed an effective simulation technique to predict the temperature effect of bridges accurately. In order to improve the computational efficiency, the substructure method was applied to the mechanical or coupled thermodynamic analysis. In addition, the effects of wind speed, atmospheric environment, and surface thermal characteristics are also considered. Wang et al. [14] separated the change of cable force caused by temperature from that caused by traffic load by monitoring the structural temperature, which provided a basis for the safety evaluation of such bridges. Structures requiring long-term construction usually undergo significant temperature changes, which will lead to the completion of the route deviating from expectations and, even in some cases, lead to construction and use failure. The research of Wang et al. [15] shows that the influence of temperature change during construction is realized by influencing the stress-free state of the structure.

The thermal effect of a cable-stayed bridge significantly influences its mechanical properties. According to the monitored temperature and displacement data, Yang et al. [16] studied the time-varying law of temperature field distribution and tower displacement of bridges. The study found that the displacement -time curve of the tower is similar to the sine curve on a typical day. The high-frequency components in the displacement -time curve can be attributed to wind and traffic load's dynamic effects. Most of the existing bridge thermal analysis studies ignore the influence of wind on the synthetic temperature distribution, and all structural surfaces adopt constant wind speed. Huang and Zhu [17] proposed a thermal analysis method to predict the temperature characteristics of steel box girder based on the actual wind field distribution around the steel box girder and studied the temperature distribution characteristics of the Honghe Bridge steel box girder. Costa et al. [18] established an accurate and robust geometrically exact formulation for nonlinear analysis of suspension structures, including geometric nonlinearity, material nonlinearity, temperature effects, and initial suspension chain line shape of suspension cables, considering the importance of temperature effects on diagonal cable structures. Thermal performance analysis, such as thermal stress calculation based on monitoring data, is very important for structural safety assessment. These temperature characteristics are more complex due to, large-span bridges temperature distribution, structural configuration, and boundary conditions. Xia et al. [19] proposed a method for structural thermal performance analysis by processing and analyzing long-term monitoring data and appl saying it to study large span suspension bridges thermal and mechanical behavior under daily operating conditions. As the primary load-bearing member of cable-stayed bridges, whether the condition is good or not always affects the bridge safety. The optimal cable is essential for prolonging the service life of the bridge, Atmaca et al. [20, 21, 22] took a pedestrian cable-stayed bridge as the research object. They proposed a new method and 3D model for the first time to optimize the cable size and post-tensioning cable force, integrating SAP2000 and MATLAB code with Open Application Programming Interface (OAPI) properties and named Jaya's metaheuristic algorithm. This model considers all service loads and determines the les' optimal size and post-tensioning force.

It is not clear how and to what extent changes in ambient temperature affect the dynamic properties of cableways in bridge engineering. Under extreme temperature conditions, the thermal conductivity of steel cables is much better than that of concrete, resulting in faster temperature changes in cable-stayed bridges than in concrete. As a result, there is a temperature difference between the base of the steel cable and the surface of the concrete beam, thereby creating additional tensile or compressive stresses in the cable that are not usually considered by practicing engineers when performing standard finite element analyses. Studies have shown that the thermal effects on the cable forces during extreme weather events (i.e., simulation results based on field measured temperatures) are more detrimental than those during average weather (i.e., simulation results based on code specified temperatures), especially when the beams exhibit positive temperature gradients.

In addition, in the two-dimensional heat transfer analysis under solar radiation, a large temperature gradient will be ignored due to the occlusion effect. In addition, the change of cable temperature is significant for the displacement caused by bridge temperature. Therefore, it is necessary to monitor the cable temperature and take measures to strengthen the insulation performance of the protective layer. During daytime traffic, the peak stress caused by elevated temperature is equivalent to that under live load, indicating that the service life of bridges is usually overestimated if the combination of live load and thermal stress is ignored.

Existing researches mainly focus on cable-stayed bridges and conventional tied arch bridges. These researches have outstanding contributions to understanding the influence of thermal effect on the static and dynamic behavior of cable or cable-stayed structures. The influence of ambient temperature on the frequency of cables is the change of cable tension and the change of cable sag. The sagging effect plays a vital role in the dynamic performance of cables. Therefore, the relationship between cable tension, sag, ambient temperature, and frequency is complex.

In addition to natural disasters such as flash floods and other extreme weather events that cause damage to bridges [23], Zhong et al. [24, 25, 26] studied the damage caused by earthquake hazards and impact loads on bridge structures. The accumulation of large amounts of wood debris can exacerbate the scouring of bridge piers, which can lead to structural damage [27], and river migration is a geomorphic hazard that affects bridge infrastructure [28]. These macroscopic high stresses on the safe usage of bridge constructions have been fruitfully explored. We need to concentrate on the impact of changes in environmental conditions, such as temperature variations, which are not clearly visible, on the safe functioning of bridges. The substantial damage to bridges caused by these temperature variations has to be examined.

This paper studies the temperature effect of a special-shaped tied-arch bridge. This kind of bridge has a complex structure and pronounced spatial effect. The finite element software is used to establish the spatial finite element model of the whole bridge. Combined with the measured temperature and cable force data, the influence of temperature load on the cable force of the bridge is analyzed. Studies have shown that the temperature sensitivity of the cables at different locations of the bridge is different, and the cable force is most significantly affected by the temperature difference of the structure. The conclusions of this paper can provide a reference for the design and maintenance of similar bridge structures.

## 2 Derivation of cable motion equation

**Figure 2:**
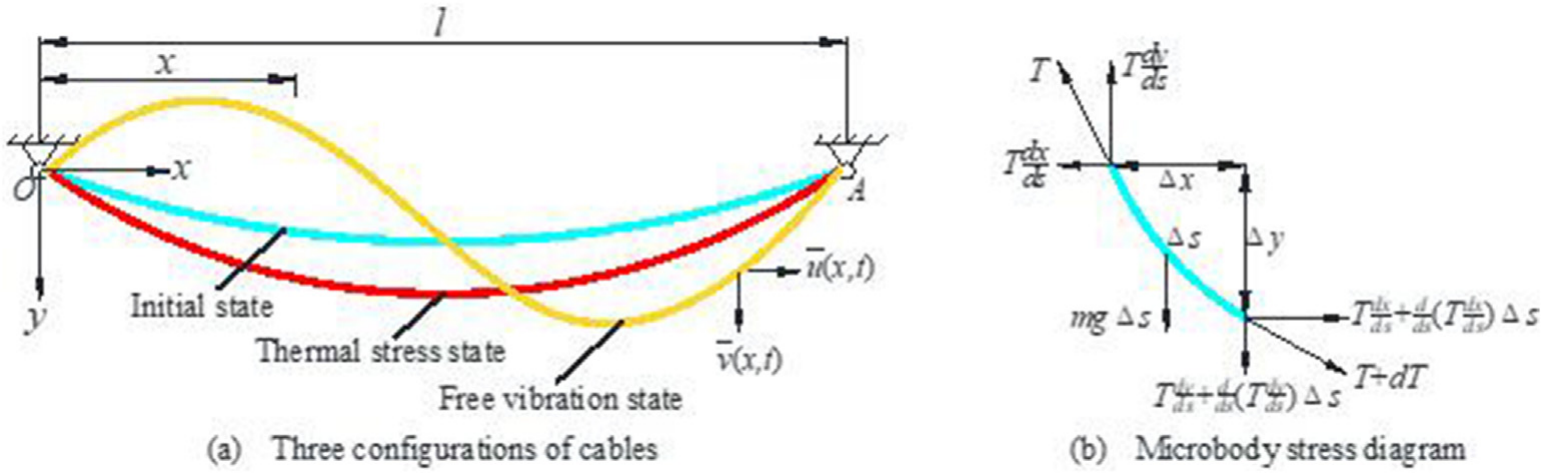
Mechanical model of cable and its stress diagram.

The mechanical model of the cable is shown in Fig.2(a) and (b). In order to simplify the calculation, the geometric and physical properties of the cable are assumed as follows:

(1) The parabola is the sag-span ratio of cable δ < 1/8 and its linear form.
(2) Ignoring the axial vibration of the cable.
(3) Ignoring the bending and shear stiffness of cables.
(4) The temperature is uniformly distributed along the cable length and cross-section, and the elastic modulus of the cable does not change with temperature.

In Figure 2, *O* is the coordinate origin, *the OA* axis is the *x*-axis, and *the y* -axis is perpendicular to *the OA* axis. The positive direction is shown in Figure 2. In the figure, *l* is the cable span, *T* is the tangential tension of the cable, and *m* is the mass of the unit length of the cable.

### 2.1 Initial state of cables

The equilibrium equations in *x* and *y* directions can be obtained by analyzing the force of the cable microelement:

(1) $$T\frac{dx}{ds}=T\frac{dx}{ds}+\frac{d}{ds}\left(T\frac{dx}{ds}\right)\Delta s$$

(2) $$T\frac{dy}{ds}=T\frac{dy}{ds}+\frac{d}{ds}\left(T\frac{dy}{ds}\right)\Delta s+mg\Delta s$$

It can be seen from Eq. (1) that T(dx/ds) is a constant. That is, the horizontal component of the cable axial force is equal everywhere, and it is set as *H*. Combined with the cable boundary conditions (${y|}_{x=0}={y|}_{x=l}=0$), the cable line can be expressed in parabolic form as shown in Eq. (3) (See the Appendix for a detailed derivation process).

(3) $$y\left(x\right)=\frac{mg}{2H}\left(l-x\right)x$$

### 2.2 Temperature stress state of cables

In order to study the influence of temperature change on the cable force and frequency, Zhao et al. [6] considered the temperature effect based on the initial state of the cable. Assuming that the temperature change is Δ*t*, the equilibrium equation of the cable is shown in Eq. (4):

(4) $$\left\{ \begin{array}{l} \frac{\partial }{\partial s}\left[\left(T+T^{\prime }\right)\left(\frac{dx}{ds}+\frac{\partial u}{\partial s}\right)\right]=0\\ \frac{\partial }{\partial s}\left[\left(T+T^{\prime }\right)\left(\frac{dy}{ds}+\frac{\partial v}{\partial s}\right)\right]=-mg \end{array}\right.$$

where: *u* and *v* are the displacements in the *x* and *y* directions of the ties due to temperature changes, respectively. *T′* is the increased tension in the ties ($T^{\prime }dx/ds=h$).

By introducing the temperature change Δ*t*, the cubic dimensionless equation of the tension increment *h* can be obtained as shown in Eq. (5).

(5) $$h^{3}+\left(2+\beta +\frac{\lambda^{2}}{24}\right)h^{2}+\left(1+2\beta +\frac{\lambda^{2}}{12}\right)h+\beta =0$$

In which $\lambda^{2}=\frac{m^{2}g^{2}L^{3}}{H^{3}}\frac{EA_{eq}}{L_{e}}$; $\beta =\alpha \Delta tL_{t}\frac{EA_{eq}}{HL_{e}}$; $L_{e}=\int_{0}^{L}{\left(\frac{ds}{dx}\right)}^{3}dx\approx L\left[1+\frac{1}{8}{\left(\frac{mgL}{H}\right)}^{2}\right]$; $L_{t}=\int_{0}^{L}{\left(\frac{ds}{dx}\right)}^{2}dx\approx L\left[1+\frac{1}{12}{\left(\frac{mgL}{H}\right)}^{2}\right]$

### 2.3 Free vibration state of cables

As shown in Figure 2, the differential equation of motion is obtained as shown in Eq. (6) in the free vibration state, neglecting the effect of lasso damping [6].

(6) $$\left\{ \begin{array}{l} \frac{\partial }{\partial s}\left[\left(T+T^{\prime }+\bar{T}\right)\left(\frac{dx}{ds}+\frac{\partial u}{\partial s}+\frac{\partial \bar{u}}{\partial s}\right)\right]=m\frac{\partial^{2}\bar{u}}{\partial t^{2}}\\ \frac{\partial }{\partial s}\left[\left(T+T^{\prime }+\bar{T}\right)\left(\frac{dx}{ds}+\frac{\partial v}{\partial s}+\frac{\partial \bar{v}}{\partial s}\right)\right]=m\frac{\partial^{2}\bar{v}}{\partial t^{2}}-mg \end{array}\right.$$

Where: $\bar{u}$ and $\bar{v}$ are the displacements of the cable in the *x* and *y* directions of plane motion, respectively. $\bar{T}$ is the increase in dynamic tension due to the vibration of the cable.

The vibration modes of the ties are divided into symmetrical and antisymmetrical modes, with the antisymmetrical modes corresponding to the vibration frequencies of the ties as shown in Eq. (7).

(7) $$\tilde{\omega }=2n\pi ,\left(n=1,2,3\cdots \right)$$

where: $\tilde{\omega }=\omega L\sqrt{m/\left(H+Hh\right)}$

The frequency corresponding to the positive symmetric mode is the solution of the following transcendental as shown in Eq. (8).

(8) $$\tan\frac{\tilde{\omega }}{2}=\frac{\tilde{\omega }}{2}-\frac{4}{{\tilde{\lambda }}^{2}}{\left(\frac{\tilde{\omega }}{2}\right)}^{3}$$

where: ${\tilde{\lambda }}^{2}=\lambda^{2}/{\left(1+h\right)}^{3}$

## 3 Bridge and monitoring system

In order to avoid the influence of traffic flow on the cable force monitoring results, this cable force monitoring was carried out under closed traffic and at different temperatures, including continuous heating and continuous cooling, for a total of eight times. Temperature measurement includes atmospheric temperature, cable temperature, arch rib temperature, and beam temperature. Atmospheric and structural temperatures are measured by air thermometer and infrared temperature measuring gun. The JMM-268 cable force dynamic measuring instrument collects the fundamental frequency of cable force.

### 3.1 Project overview

**Figure 3:**
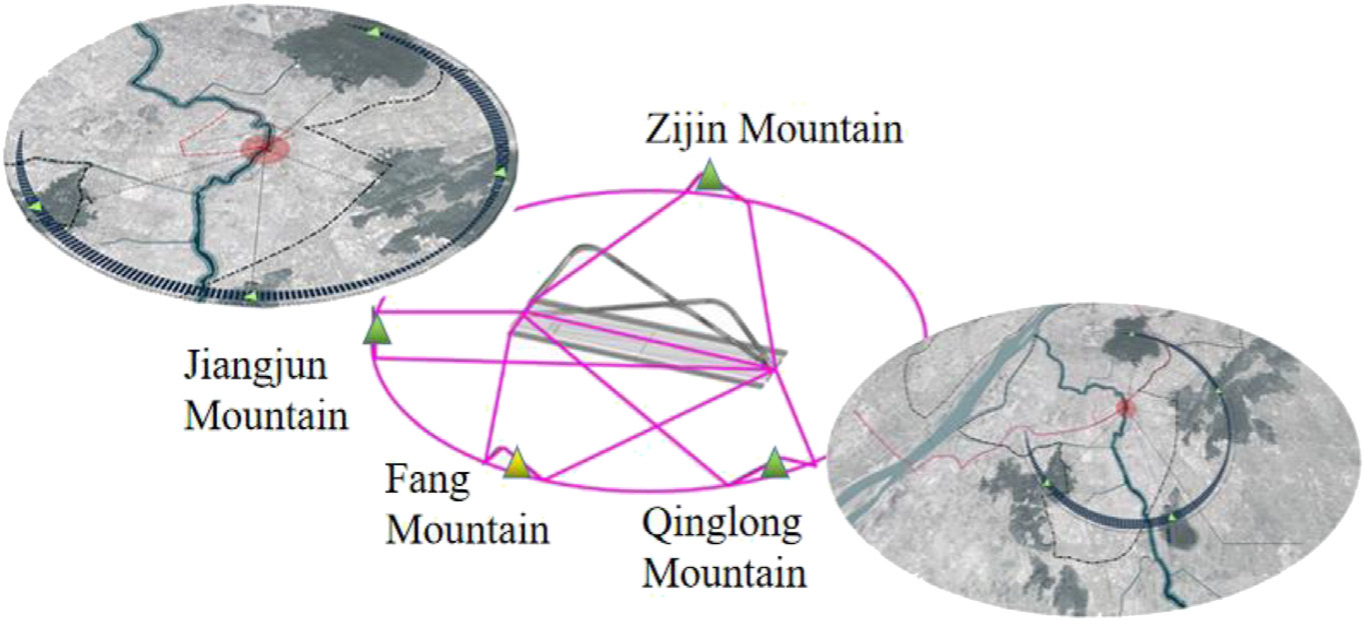
Qinhuai bay bridge.

The Qinhuai Bay Bridge in Nanjing, China, is the subject of this paper. It is a complex, tied-arch bridge that crosses the Qinhuai River. The year of design is 2018, and it was done in 2021. If looking at Figure 3, Qinhuai Bay Bridge, which spans the Qinhuai River and links Nanjing city's CBD and ecological park in the south, is a significant gateway to the city and a major urban landmark. It also connects to the Beijing-Shanghai High-speed Railway on the south side and the Nanjing city bypass on the north. The functional needs and the surroundings of this bridge are very complicated. The line has to cross highways and rivers and go under an elevated railroad. It has to handle all kinds of traffic, including motorized, non-motorized, and pedestrian. The Qinhuai Bay Bridge has two parts. The Qinhuai Bay Bridge is 157 m long and 42 m wide, with a two-way, six-lane motorway that goes both ways. “Duplex structure”: The outermost non-motorized lanes and sidewalks on the bridge are two separate parts, one above the other. A sidewalk is on top, and a non-motorized lane is below.

Note that Nanjing has a humid subtropical climate with only two seasons, cold and hot years, which is vital to know. Living in Nanjing might think of it as a “furnace” because it boils in July and August. Nanjing is the first city in southern China to get very cold in the winter, with a low temperature of 7.7–13.3 degrees Celsius.

**Figure 4:**
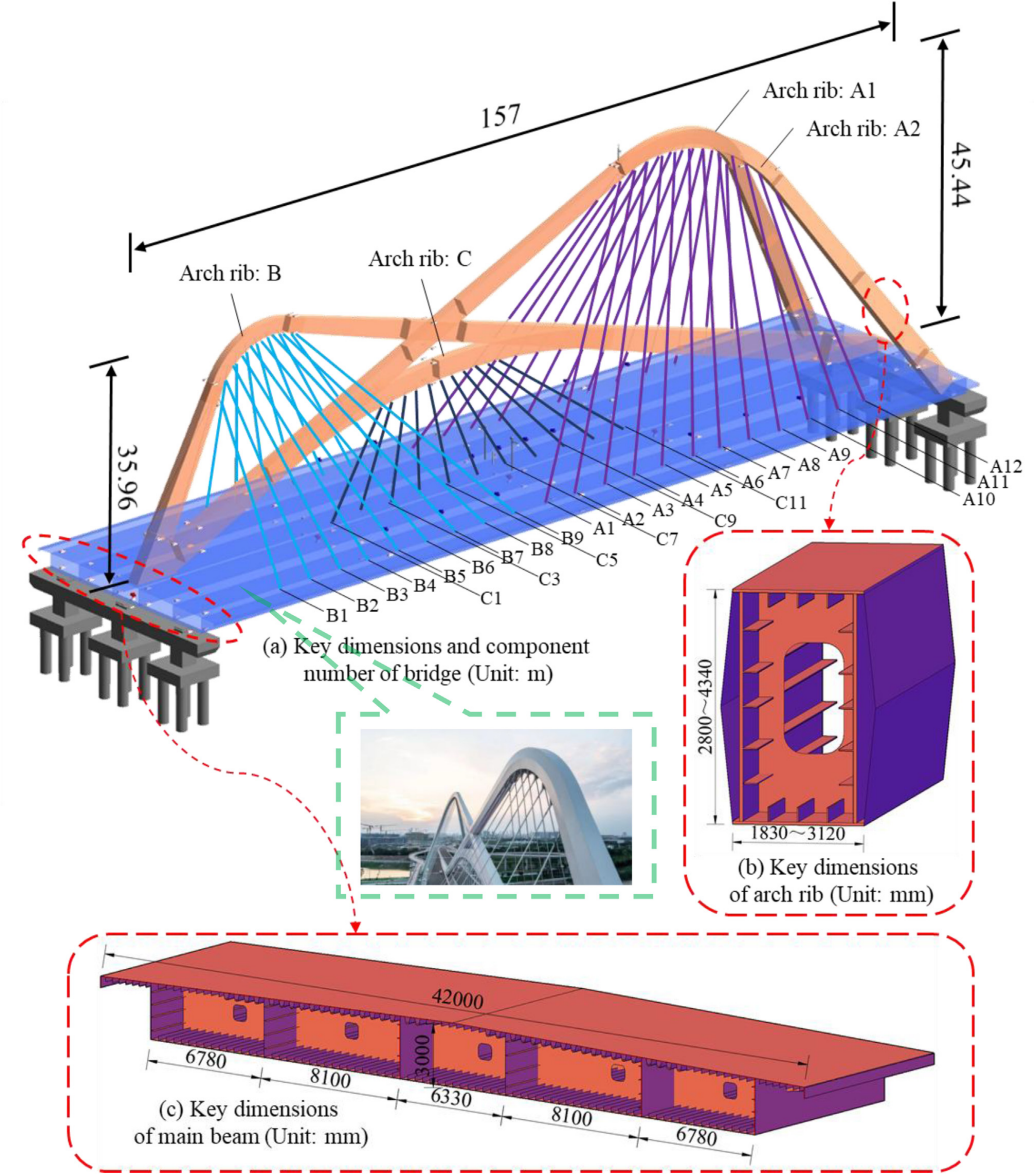
Overview of nanjing qinhuai bay bridge.

The spatial distribution of the Nanjing Qinhuai Bay Bridge is shown in Figure 4. The bridge is 157 m long, with a girder width and height of 42 m and 3 m, respectively. The tie beam is a flat steel box girder with a single box and five chambers, and the arch ribs are steel box arches with a unique spatial arrangement, in which arch rib B and arch rib C are arranged along the longitudinal bridge axis, and arch rib A bifurcates at the top of the arch to form a “Y -shaped” arrangement.

All cables are inclined and distributed, with 53 cables in total. Depending on the forces, the cables consist of a new unstressed LZM7-55/61 outer layer and anchorage devices at both ends. The individual cables consist of Φ 7 galvanized high-strength low relaxation prestressing steel wires with a standard strength of 1 670 MPa. For the convenience of subsequent discussion, the ties are numbered, with the suitable widths being B1 to B9 and A1 to A12, respectively, the left widths being B1′ to B9′ and A1′ to A12′, respectively, and the ties located within the central parting zone being numbered C1 to C11.

### 3.2 Temperature field and rope force monitoring

**Figure 5:**
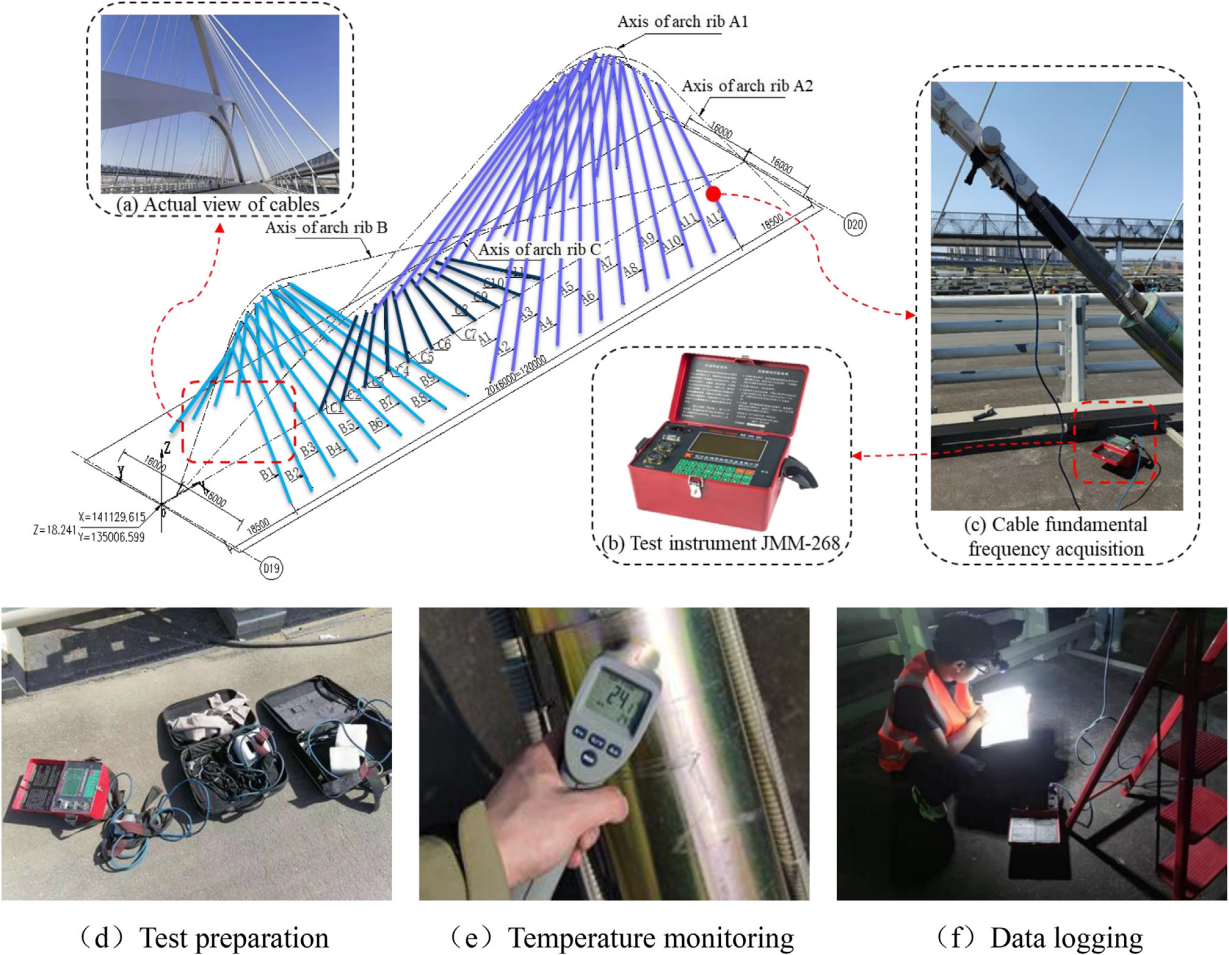
Field testing of Nanjing Qinhuai Bay Bridge.

The field test is shown in Figure 5. The rigging tests were carried out in closed traffic and at different temperatures, including continuous warming and continuous cooling, a total of eight times. Temperature measurements include the atmosphere, cables, arch ribs, and beams. Temperatures of the atmosphere and structures are determined using an air thermometer and an infrared thermometer. A JMM-268 cable force dynamic tester is used to determine cable force.

Since the bridge studied in this paper is a unique bridge with multiple arch ribs and multiple cable faces, the ties at arch ribs A, B, and C were selected for the study in order to provide a more comprehensive response to the effect of temperature changes on the cable force of the bridge. In addition, related studies [29] showed that the side and middle cables of tied-arch bridges are more sensitive to temperature changes, so this factor was considered in the selection of test cables. In summary, the test ties were selected as follows: arch ribs A: A1, A1′, A6, A6′, A12, A12'; arch rib B: B1, B1 ′, B5, B5 ′, B9, B9 '; arch rib C: C1, C5, C6, C11, a total of 16 cable test.

### 3.3 Analysis of temperature field monitoring data

**Figure 6:**
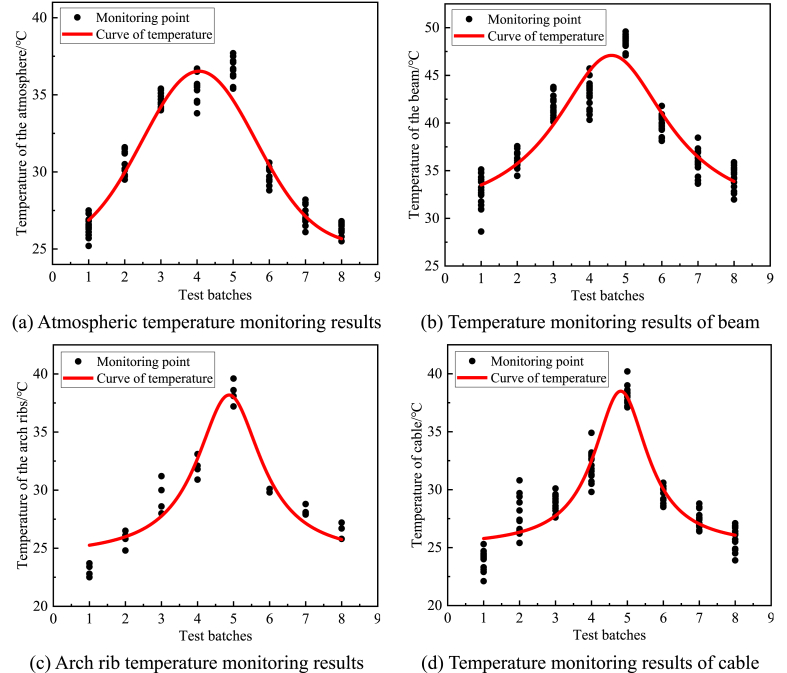
Temperature field monitoring results (a) Atmospheric temperature monitoring results (b) Temperature monitoring results of beam (c) Arch rib temperature monitoring results (d) Temperature monitoring results of cable.

The results of temperature field monitoring are shown in Figure 6. The red line in the figure is obtained by fitting a Gaussian function. It can be seen from Figure 6(a)–(d) that in this cable force test, the temperature of the arch rib and cable body is relatively close, and the average temperature difference is between −2 °C and +1 °C. The maximum temperature of the arch rib is 38.4 °C on the day, while the temperature difference between beam and arch is significant, and the average temperature difference is +9.8 °C. The maximum temperature of the beam body is 48.5 °C on the day, which is due to the different parts of the bridge structure being affected by sunshine and temperature. When the bridge is directly exposed to sunlight, compared with the appearance of white arch ribs and cables, the surface of the beam is more straightforward to absorb heat due to the covering of a dark asphalt pavement layer, and the interior of the beam is nearly closed and not easy to heat dissipation, which leads to a significant temperature difference between the structures. In addition, the temperature change rates of various parts of the bridge are also different. The temperature of each part of the bridge varies nonlinearly, while the arch rib and cable body temperature has a strong nonlinearity compared to the beam temperature. Therefore, the temperature difference between the structures with different temperature rise and fall rates will be generated at the same time.

### 3.4 Analysis of cable force monitoring data

The principle of the vibration frequency method is to install the sensor on the cable and excite the cable by manual or environmental means, the sensor then collects its acceleration signal and analyzes it, draws the spectrum, and obtains the vibration frequency. Then the cable force is calculated by the relationship between the cable force and the self-oscillation frequency. By simplifying the cable as a bending vibration model with axial force on the hinged beam at both ends, the current widely used cable force calculation equation is obtained as shown in Eq. (9).

(9) $$T=\frac{4ml^{2}f_{n}^{2}}{n^{2}}-\frac{n^{2}\pi^{2}}{l^{2}}EI$$

**Figure 7:**
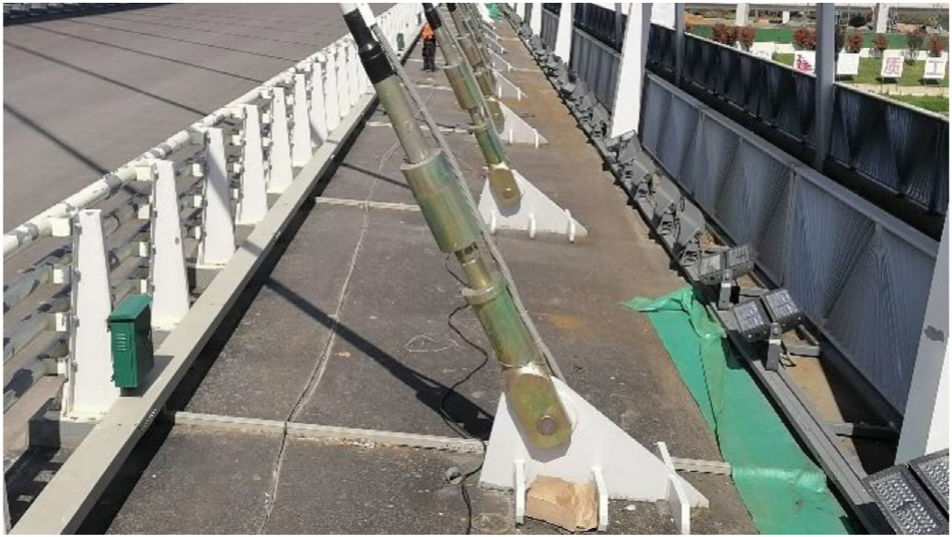
End structure of cable.

Unlike the conventional cable anchorage form, the bridge cable under study in this paper is pin-hinged anchorage, as shown in Figure 7.

The cable boundary conditions under this anchorage method are complex, and the current widely used cable force calculation formulae cannot meet the needs of such complex boundary conditions in the application of this project, so scholar Shen Kun [30] took the bridge in this case as the research object, proposed explicit expressions for complex boundary conditions and verified each other with finite elements, and formulas proposed by other scholars to provide a formula basis for cable force testing as shown in Eq. (10).

(10) $$T=\left\{ \begin{array}{cc} 4ml^{2}\left(\frac{-1.53}{l}+0.98\right)f^{2} & \left(\xi >40,0<T\leq 500kN\right)\\ 4ml^{2}\left(\frac{-0.28}{l}+0.98\right)f^{2} & \left(\xi >40,T>500kN\right)\\ 4ml^{2}\left(\frac{6.68}{l}+0.95\right)f^{2} & \left(\xi \leq 40\right) \end{array}\right.$$

Note: *T* - cable force; *m* - linear density; *l* - theoretical calculated length; *f* - fundamental frequency; *ξ* - fundamental frequency.

The results of the measured cable frequencies are shown in Table 1.

**Table 1:** Test results of cable frequency.

No.	Cable No.	Theoretical calculation length/m	Base frequency/Hz							
			Batch 1	Batch 2	Batch 3	Batch 4	Batch 5	Batch 6	Batch 7	Batch 8
1	A1	49.282	2.058	2.041	2.053	2.047	2.054	2.054	2.067	2.053
2	A1′	49.290	2.052	2.039	2.041	2.038	2.038	2.033	2.05	2.053
3	A6	44.162	2.444	2.434	2.411	2.413	2.419	2.434	2.441	2.458
4	A6′	44.186	2.423	2.412	2.393	2.414	2.417	2.427	2.429	2.434
5	A12	34.494	2.874	2.846	2.834	2.833	2.807	2.842	2.844	2.858
6	A12′	34.500	2.836	2.819	2.805	2.797	2.82	2.838	2.862	2.85
7	C1	15.804	6.786	6.683	6.674	6.618	6.413	6.534	6.675	6.711
8	C5	14.605	6.965	6.932	6.845	6.776	6.461	6.759	6.784	6.838
9	C6	15.930	6.55	6.547	6.456	6.316	6.235	6.434	6.471	6.502
10	C11	28.045	3.647	3.606	3.576	3.543	3.468	3.557	3.585	3.609
11	B1	27.147	3.794	3.778	3.768	3.762	3.745	3.778	3.792	3.808
12	B1′	27.182	3.807	3.79	3.783	3.856	3.844	3.809	3.839	3.816
13	B5	37.072	2.681	2.717	2.68	2.72	2.718	2.721	2.727	2.728
14	B5′	37.100	2.762	2.746	2.737	2.746	2.772	2.756	2.758	2.757
15	B9	45.200	2.131	2.115	2.133	2.123	2.142	2.148	2.149	2.128
16	B9′	45.237	2.222	2.216	2.22	2.218	2.239	2.23	2.236	2.243

**Figure 8:**
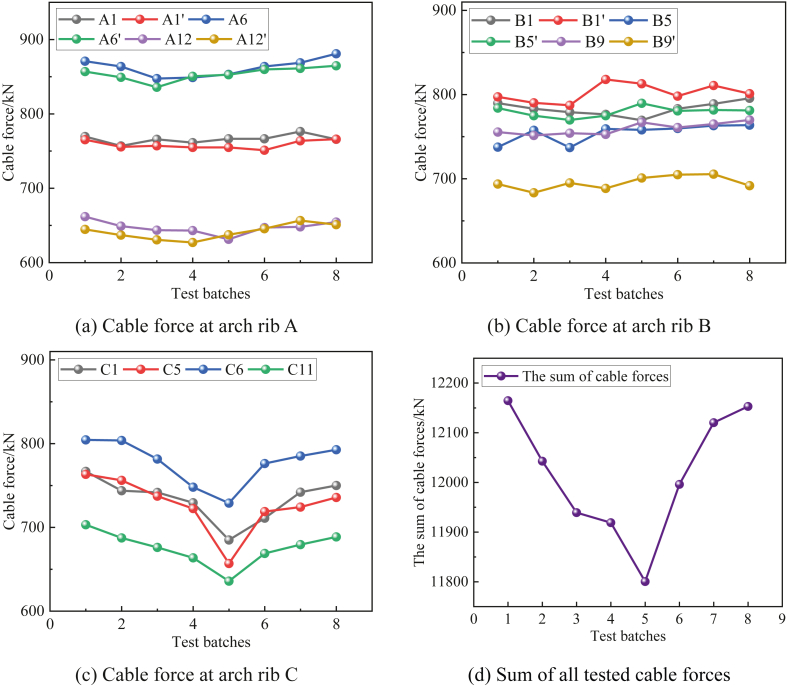
Cable force test results (a) Cable force at arch rib A (b) Cable force at arch rib B (c) Cable force at arch rib C (d) Sum of all tested cable forces.

A total of 16 cables were selected in this cable force test, including six cables at arch ribs A and B and four cables at arch ribs C. As shown in Figure 8(c), The test cable force varies with the change in temperature. The trend of the force at arch rib C is inversely proportional to the temperature change; that is, when the temperature increases, the force decreases, and when the temperature decreases, the force increases, and this relationship is pronounced. In contrast, from Figure 8(a) and (b), the force at arch ribs A and B showed a fluctuation with temperature. In addition, it can be seen from Figure 8(d) that the total cable force of the 16 cables in this test is also inversely proportional to the temperature.

**Figure 9:**
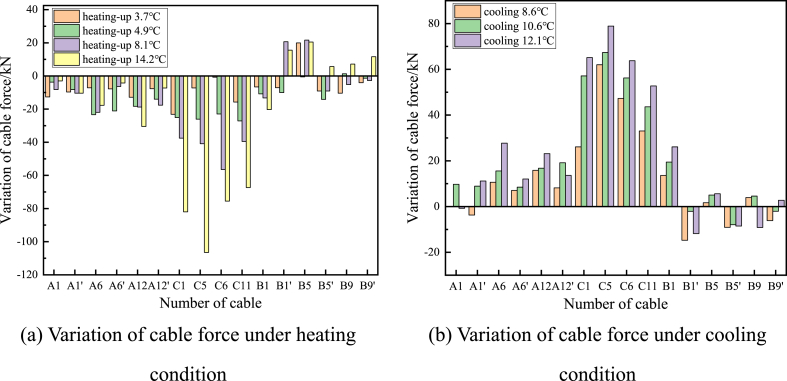
Cable force change with temperature (a) Variation of cable force under heating condition (b) Variation of cable force under cooling condition.

The measured data are divided into continuous heating and continuous cooling for comparative analysis, and the influence of temperature rise and fall on cable force is discussed. From Figure 9(a), and (b), it can be seen that under the condition of monotonous temperature change, the change of cable force in different parts is inconsistent, and the inconsistency is mainly reflected in the inconsistent change trend and the inconsistent change amplitude.

The changing trend is not consistent; with the increase of temperature, not all the cable tensions decrease, but there are two states of partial cable tension increase and cable tension decrease, and the opposite trend changes in the case of temperature decrease. This is because the change of the cable force is not only affected by the thermal expansion and contraction of the cable itself, but also the uncoordinated deformation of the beam arch caused by the temperature difference effect between the structures will change the relative position of the two ends of the cable, thus affecting the cable force. The inconsistent variation amplitude of cable force is reflected by the different variations of cable force at different positions under the same temperature. The variation of cable force at arch rib C is the largest, and the variation of cable force at 14 °C is up to −106.5 kN, with a change rate of −13.9 %. The variation of cable force at arch ribs A and B is small and close. When the cable is also heated at 14 °C, the maximum variation of cable force is only −30.5 kN, with a change rate of −4.6 %, indicating that the temperature sensitivity of cables at different positions is quite different.

This related to the particular special arrangement of cable faces and the wide flat beam. The bridge is 42 m wide and has three cable faces in the cross-bridge direction (with the C-arch rib cable face in the central divider and the remaining two cable faces symmetrically arranged in the left and right side dividers), which can simplify the main beam transversely to a three-point support state. When the temperature causes the rise of the cable force on both sides, the cable connecting the C-arch will distribute less cable force, resulting in a sharp drop of the cable force. The cable connecting the C-arch will distribute more force in the opposite case. Therefore, the force variation of the cables at arch rib C is larger than that of the cables at arch ribs A and B.

In addition, the maximum amplitude of cable force change at arch rib C is related to the relative cable length and the flexural stiffness of the arch rib, and the average cable lengths of the booms at arch ribs A, B, and C are 42.9 m, 36.8 m, and 18.4 m. To meet the deformation coordination, the same amount of cable length change causes different values of cable force change, and the short cable will produce a larger cable force change than the long cable. Therefore, the change in cable force of the boom at arch rib C is the largest.

## 4 Simulation of cable force thermal effect based on measured temperature field

In the design of bridge structures, it is often necessary to consider the influence of temperature effects on the structure, which are specified in the bridge design codes of various countries. Although there are differences in the values of temperature loads in the codes of various countries, the types of temperature loads, in general, include the overall temperature difference and the temperature gradient of the beam section. Bridges are subject to sunlight angle, ventilation conditions, and differences in material properties that can cause significant temperature differences between structures. The internal structural forces caused by the temperature difference are not even less than the traffic load. In this paper, FEA software will simulate three temperature effects to analyze the effect of temperature on the rigging force. The overall temperature difference and the beam section temperature gradient are input in the model using the code specified values, while the structural temperature difference is input in the model using the measured temperature field data. In order to save computational resources, the temperatures of the arch ribs, cables, and beams are considered to be uniformly distributed when simulating the structural temperature differences, but there are temperature differences between the three.

### 4.1 Finite element modeling

**Figure 10:**
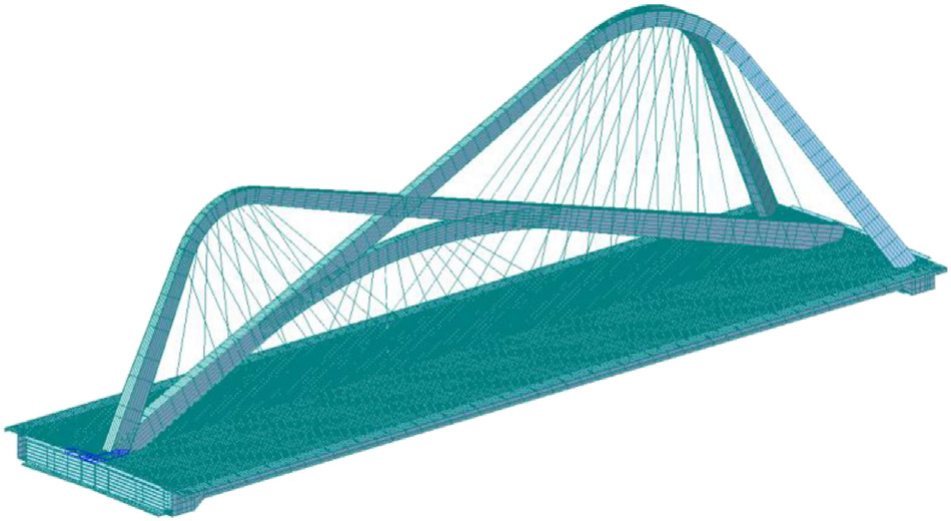
Finite element model.

The finite element analysis software is used to establish the spatial model of the whole bridge. Figure 10 depicts a finite element model of the system. The beam element is utilized while simulating the arch rib and main beam, and the truss element is used when simulating the cable. There are 705 nodes and 985 elements in the model. Table 2 contains a list of the significant modeling parameters.

**Table 2:** Material and section geometric characteristics.

Components	material type	Cross -sectional area/m^2^	Moment of inertia of cross -section/m^4^	Elastic modulus/GPa	Unit weight/kN·m ^−3^
Tie beam	Q345 steel	2.281	3.782	206	76.98
Arch ribs	Q345 steel	0.387–0.684	0.490–1.336	206	76.98
Cable	Φ7mm Steel wire	0.011	0.000	195	78.50

### 4.2 Thermal effect analysis of cable force

#### 4.2.1 Bridge overall temperature effect

With the seasonal change, the ambient temperature also changes, but this change process lasts for a long time and changes slowly. When the seasonal temperature change affects the structure, it can be approximately considered that the resulting structural temperature change is uniform. When considering the overall temperature change of the structure, it is usually based on the annual temperature change value. The annual temperature change value refers to the average temperature of the highest month in one year minus the average temperature of the lowest month. When the overall temperature change acts on the structure without horizontal constraints, the internal fiber is uniformly stretched and does not produce temperature secondary internal force. The temperature stress caused by the overall temperature change cannot be ignored for statically indeterminate structures such as tied-arch bridges and cable-stayed bridges.

**Figure 11:**
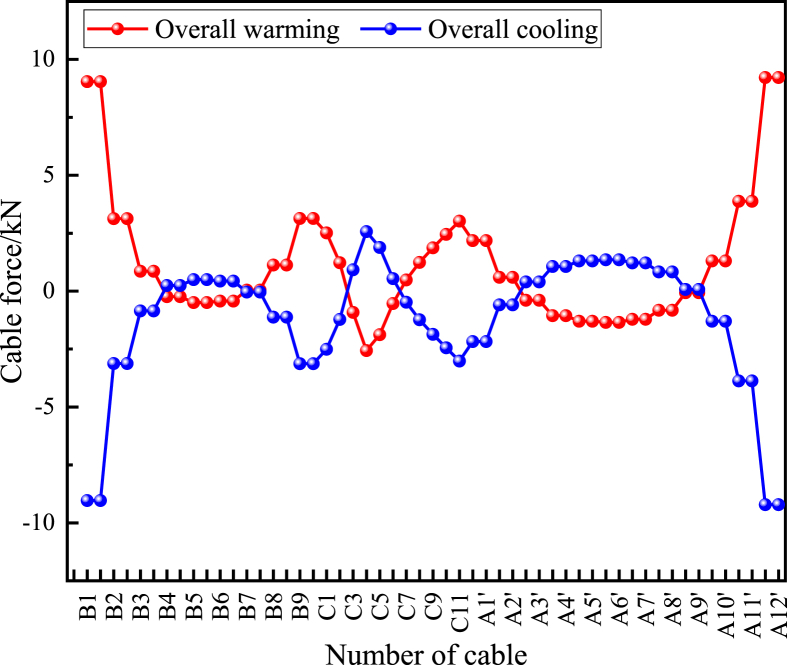
Cable force variation under the overall temperature effect.

According to the bridge design document of this example, when calculating the overall temperature effect, the rise and fall of the whole bridge are considered at + 25 °C and −25 °C, respectively (the reference temperature is 15 °C), and the change of the cable force under the overall temperature is analyzed. It can be seen from Figure 11 that the variation trend of cable force is opposite when the overall temperature rises and falls, and the variation range of cable force is within ±10 kN. Among them, the longitudinal end cable of the bridge is affected by the overall temperature rise and fall. The maximum cable force variation of A12 cable is ±9.2 kN, and the change rate is ±1.3 %. In addition, under the action of overall temperature, the cable force does not all rise or fall, reflecting the inconsistency of the cable force change trend.

#### 4.2.2 Temperature gradient effect of steel box girder section

The bridge structure in the natural environment will be affected by solar radiation, ground reflection, air flow change, and other factors, which will lead to uneven distribution of temperature fields inside the structure. Due to the direct sunlight irradiation on the sunny side of the structure, the structure's surface will rapidly increase temperature, and the conduction of temperature takes time, which leads to the lag of temperature change inside the structure. For example, the back of the beam is not directly radiated by the sun, the ventilation condition is usually better, and the surface temperature is even slightly lower than the atmospheric temperature, which will aggravate the temperature difference between the top and bottom of the beam.

**Figure 12:**
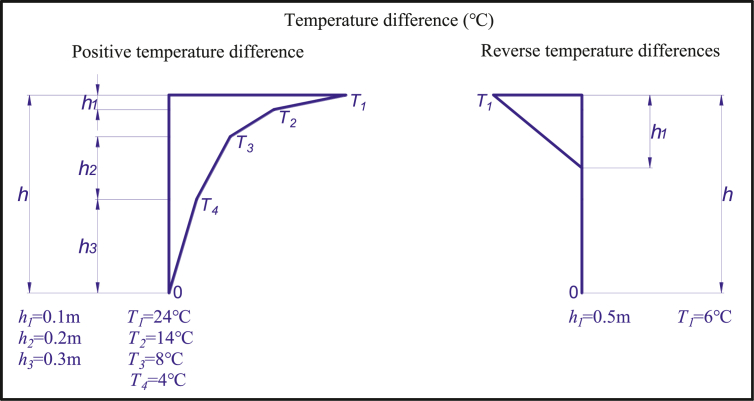
British standard BS -5400 temperature gradient model.

Due to the good thermal conductivity of steel, the steel box girder is highly sensitive to temperature change, so it is necessary to study the influence of the vertical temperature gradient of the steel box girder section on the cable force of this bridge. The bridge deck pavement of this case is 40mm modified asphalt SMA +40mm pouring asphalt concrete. The vertical temperature gradient of the main beam is used according to the British BS -5400 specification, and the specific value is shown in Figure 12.

**Figure 13:**
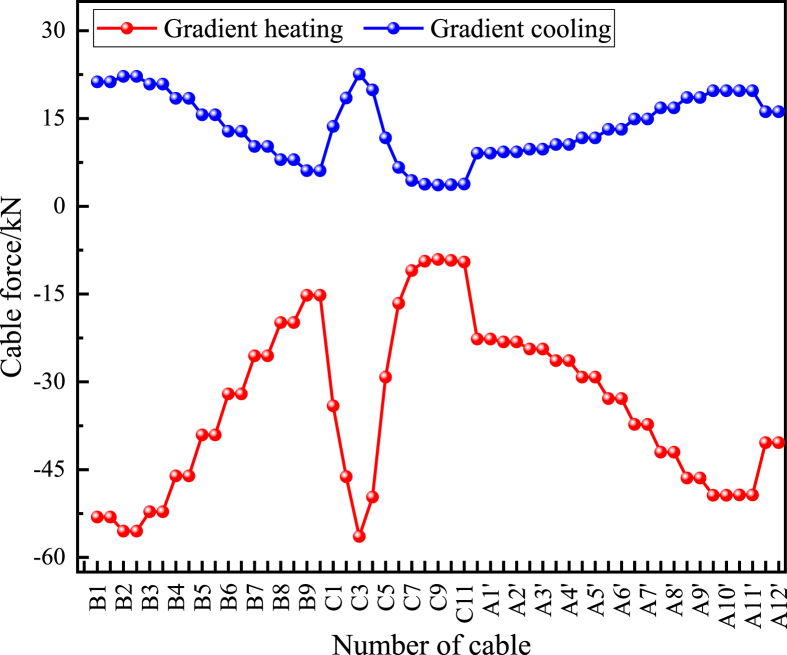
Variation of cable force under temperature gradient.

Unlike the overall temperature effect, the cable force under the gradient temperature rise and fall of the main beam tends to decrease or increase, but the change varies. From Figure 13, it can be seen that the change of cable force under the gradient warming effect of the main girder ranges from -56.4 to -9.1 kN, and the change of cable force under the gradient cooling effect ranges from 3.6 to 22.6 kN. This value indicates that the effect of the gradient temperature of the main girder on the cable force is greater than that of the overall temperature effect. In addition, the longitudinal bridge end ties are more sensitive to the temperature gradient effect of the main beam.

#### 4.2.3 Temperature difference effect of structure

There are differences in the spatial location, size, shape, appearance coating, and material properties of each bridge member, so different parts of the bridge have different temperature sensitivity, which can lead to temperature differences when each bridge member is subjected to temperature action, resulting in temperature stresses in the structure.

This paper combines the measured temperature field data and establishes four temperature difference load conditions, respectively, the arch ribs and tension cables are 4 °C higher than the main beam (condition 1), 6 °C (condition 2), 8 °C (condition 3), and 10 °C (condition 4), to analyze the effect of temperature difference effect between structures on the tension cable force.

**Figure 14:**
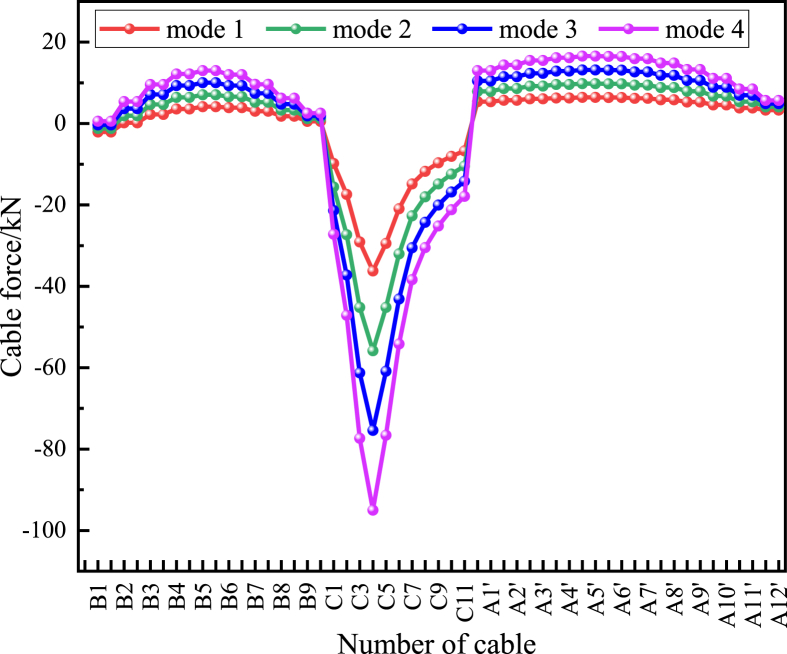
The variation value of cable force under a structural temperature difference.

From Figure 14, it can be seen that under the effect of structural temperature difference, the cable force at arch ribs A and B shows an increasing trend, and the cable force at arch rib C shows a decreasing trend, and the more significant the temperature difference between beam and arch, the more noticeable this trend is. This result is in good agreement with the measured force data. It can be seen that, among many temperature effects, the structural temperature difference causes the most considerable change in the cable force, and this influence factor should be fully considered in the design of similar projects.

## 5 Conclusions

Based on cable force measurements and model calculations, this work investigates the influence of temperature change on the cable force of the space complex heterogeneous tethered arch bridge. The following findings are made.

(1) Different parts of the bridge structure are affected by sunlight and temperature to different degrees, leading to differences in the rate of temperature change of each part of the bridge. The temperature difference between the cable body and the arch rib is slight, within ±2 °C. In contrast, the temperature difference between the girder arch is significant, averaging up to +9.8 °C, and the influence of the significant temperature difference between the structures on the bridge cannot be ignored.
(2) The total cable force of the bridge is inversely proportional to the temperature rise and fall, and the temperature sensitivity of the cable at different locations is significantly different. The cable force is affected by the thermal expansion and contraction of the material itself, and the uncoordinated displacement of the beam -arch caused by the temperature will change the cable force.
(3) Temperature changes have an enormous impact on the cable force of the bridge, in this case, the maximum change in single cable force can reach -106.5 kN at 14 °C, with a change rate of -13.9%, and the change in cable force at different parts of the bridge is different under the same temperature, and the trend of increase and decrease in cable force is also inconsistent.
(4) From the spatial location, the overall temperature effect and the main beam temperature gradient effect have a more significant effect on the cable force at the end of the longitudinal bridge, and the structural temperature difference effect has a more significant effect on the cable force at the middle of the cross-bridge. From the numerical point of view, the structural temperature difference effect causes the enormous change in the cable force and is more consistent with the measured data, and the influence factor should be fully considered in the design of similar projects.
(5) The tension cable force will change significantly due to the temperature change. These ties should be paid in bridge design evaluation and operation monitoring. Also, if appropriate temperature fields are used based on bridge field measurements, the temperature-induced changes in cable forces can be accurately assessed.
(6) In the finite element modeling process, 16 sets of measured temperature field data are used in this paper to input the structural temperature difference in the model. The authors will collect more measured data to establish a more refined finite element model in future research work.

## Declarations

### Author contribution statement

Liming Zhu, Tailei Chen, Wonsun King: Performed the experiments; Analyzed and interpreted the data; Wrote the paper.

Lingkun Chen: Conceived and designed the experiments; Analyzed and interpreted the data; Wrote the paper.

Xiaojian Han: Analyzed and interpreted the data; Contributed reagents, materials, analysis tools or data; Wrote the paper.

Lu Wang, Chencheng Zhai: Performed the experiments; Wrote the paper.

Yuan Tian: Contributed reagents, materials, analysis tools or data; Wrote the paper.

### Funding statement

This work was supported by National Key R & D Program [2021YFB2600600].

### Data availability statement

Data included in article/supp. material/referenced in article.

### Declaration of interests statement

The authors declare no conflict of interest.

### Additional information

No additional information is available for this paper.

## Appendix

It can be seen from Eq. (1) that T(dx/ds) is a constant, that is, the horizontal component of the cable axial force is equal everywhere, and it is set as *H*, and the following equation can be obtained:

(11) $$T\;\frac{dx}{ds}=H$$

From Eq. (2) it follows that:

(12) $$-mg=\frac{d}{ds}\left(T\frac{dy}{ds}\right)$$

The conjunction Eq.(11) and Eq.(12) gives:

(13) $$-mg\frac{ds}{dx}=H\frac{d^{2}y}{dx^{2}}$$

From the micro -element arc length assumption, Eq. (13) can be transformed to:

(14) $$-mg\sqrt{1+{\left(\frac{dy}{dx}\right)}^{2}}=H\frac{d^{2}y}{dx^{2}}$$

Bringing in the boundary condition: ${y|}_{x=0}={y|}_{x=l}=0$, and solve Eq. (14) to obtain Eq. (15).

(15) $$y\left(x\right)=\frac{mg}{2H}\left(l-x\right)x$$
